# Genome-wide analysis of genetic correlation in dementia with Lewy bodies, Parkinson's and Alzheimer's diseases

**DOI:** 10.1016/j.neurobiolaging.2015.10.028

**Published:** 2016-02

**Authors:** Rita Guerreiro, Valentina Escott-Price, Lee Darwent, Laura Parkkinen, Olaf Ansorge, Dena G. Hernandez, Michael A. Nalls, Lorraine Clark, Lawrence Honig, Karen Marder, Wiesje van der Flier, Henne Holstege, Eva Louwersheimer, Afina Lemstra, Philip Scheltens, Ekaterina Rogaeva, Peter St George-Hyslop, Elisabet Londos, Henrik Zetterberg, Sara Ortega-Cubero, Pau Pastor, Tanis J. Ferman, Neill R. Graff-Radford, Owen A. Ross, Imelda Barber, Anne Braae, Kristelle Brown, Kevin Morgan, Walter Maetzler, Daniela Berg, Claire Troakes, Safa Al-Sarraj, Tammaryn Lashley, Yaroslau Compta, Tamas Revesz, Andrew Lees, Nigel J. Cairns, Glenda M. Halliday, David Mann, Stuart Pickering-Brown, John Powell, Katie Lunnon, Michelle K. Lupton, International Parkinson's Disease Genomics Consortium (IPDGC), Dennis Dickson, John Hardy, Andrew Singleton, Jose Bras

**Affiliations:** a Department of Molecular Neuroscience, Institute of Neurology, UCL, London, UK; b MRC Centre for Neuropsychiatric Genetics and Genomics, School of Medicine, Cardiff University, Cardiff, UK; c Nuffield Department of Clinical Neurosciences, Oxford Parkinson's Disease Centre, University of Oxford, Oxford, UK; d Laboratory of Neurogenetics, National Institutes on Aging, NIH, Bethesda, MD, USA; e German Center for Neurodegenerative Diseases (DZNE), Tübingen, Germany; f Taub Institute for Alzheimer Disease and the Aging Brain, Columbia University, New York, NY, USA; g Department of Pathology and Cell Biology, Columbia University, New York, NY, USA; h Department of Neurology, Columbia University, New York, NY, USA; i Department Of Neurology and Alzheimer Center, Neuroscience Campus Amsterdam, VU University Medical Center, Amsterdam, the Netherlands; j Department of Medicine, Tanz Centre for Research in Neurodegenerative Diseases, University of Toronto, Toronto, Ontario, Canada; k Cambridge Institute for Medical Research, and Cambridge National Institute of Health Research Biomedical Research Unit in Dementia, University of Cambridge, Cambridge, UK; l Clinical Memory Research Unit, Institute of Clinical Sciences Malmö, Lund University, Lund, Sweden; m Clinical Neurochemistry Laboratory, Department of Psychiatry and Neurochemistry, Institute of Neuroscience and Physiology, Sahlgrenska Academy at the University of Gothenburg, Gothenburg, Sweden; n Neurogenetics Laboratory, Division of Neurosciences, Center for Applied Medical Research, (CIMA), University of Navarra, Pamplona, Spain; o CIBERNED, Centro de Investigación Biomédica en Red de Enfermedades Neurodegenerativas, Instituto de Salud Carlos III, Madrid, Spain; p Memory and Movement Disorders Units, Department of Neurology, University Hospital Mutua de Terrassa, University of Barcelona School of Medicine, Terrassa, Barcelona, Spain; q Departments of Psychiatry and Psychology, Mayo Clinic, Jacksonville, FL, USA; r Department of Neurology, Mayo Clinic, Jacksonville, FL, USA; s Department of Neuroscience, Mayo Clinic, Jacksonville, FL, USA; t Translation Cell Sciences—Human Genetics, School of Life Sciences, Queens Medical Centre, University of Nottingham, Nottingham, UK; u Hertie Institute for Clinical Brain Research, Department of Neurodegeneration, Center of Neurology, University of Tuebingen, and DZNE, German Center for Neurodegenerative Diseases, Tuebingen, Germany; v MRC London Neurodegenerative Diseases Brain Bank, Institute of Psychiatry, Department of Clinical Neuroscience, King's College London, London, UK; w Queen Square Brain Bank, Department of Molecular Neuroscience, UCL Institute of Neurology, London, UK; x Parkinson's Disease & Movement Disorders Unit, Neurology Service, Clinical Neuroscience Institute (ICN), Hospital Clínic/University of Barcelona/IDIBAPS, Barcelona, Spain; y Department of Neurology, Knight Alzheimer's Disease Research Center, Washington University School of Medicine, Saint Louis, MO, USA; z Neuroscience Research Australia, Sydney, Australia; aa School of Medical Sciences, Faculty of Medicine, University of New South Wales, Sydney, Australia; bb Institute of Brain, Behaviour and Mental Health, Faculty of Medical and Human Sciences, University of Manchester, Manchester, UK; cc Department of Basic and Clinical Neuroscience, Institute of Psychiatry, Psychology and Neuroscience, King's College London, London, UK; dd Institute of Clinical and Biomedical Science, University of Exeter Medical School, University of Exeter, Exeter, UK; ee Reta Lila Weston Research Laboratories, Department of Molecular Neuroscience, UCL Institute of Neurology, London, UK

**Keywords:** Dementia with Lewy bodies, Alzheimer's disease, Parkinson's disease, Genetic correlation

## Abstract

The similarities between dementia with Lewy bodies (DLB) and both Parkinson's disease (PD) and Alzheimer's disease (AD) are many and range from clinical presentation, to neuropathological characteristics, to more recently identified, genetic determinants of risk. Because of these overlapping features, diagnosing DLB is challenging and has clinical implications since some therapeutic agents that are applicable in other diseases have adverse effects in DLB. Having shown that DLB shares some genetic risk with PD and AD, we have now quantified the amount of sharing through the application of genetic correlation estimates, and show that, from a purely genetic perspective, and excluding the strong association at the *APOE* locus, DLB is equally correlated to AD and PD.

## 1 Introduction

As we move toward an era where precision medicine becomes a reality, being able to confidently differentiate between closely related diseases is fast becoming a key priority. This is even more relevant when therapeutic approaches from one disease have negative effects when used in patients from another, as is the case in dementia with Lewy bodies (DLB) where neuropsychiatric and dysautonomic features can be worsened by dopaminergic agents used in Parkinson's disease (PD; Zweig and Galvin, 2014).

DLB is probably one of the most underserved common disorders and much of this stems from the fact that it is a disease for which a clinical diagnosis is a particularly difficult one to make as DLB can be misdiagnosed as Alzheimer's disease (AD) when starting with cognitive impairment or as PD when presenting with parkinsonism, and in turn PD can be easily mistaken as DLB if parkinsonism is overlooked. There are numerous shared aspects between DLB and the other more common neurodegenerative diseases PD and AD. This is not only true at the clinical level (particularly in the case of DLB and PD, to the point that an artificial and arbitrary “one-year-rule” in terms of the timing between parkinsonism and dementia has been needed to delineate them), but also, to some extent, at the pathological level, where Lewy bodies are a common characteristic of both DLB and PD, and beta-amyloid plaques and tau-positive neurofibrillary tangles, hallmarks of AD, often coexist in DLB and PD brains leading to the suggestion of a synergism between these pathologies (Compta et al., 2011, McKeith et al., 2005).

It is key that we have a better understanding of the molecular mechanisms occurring in DLB, not only because this is pivotal information for novel therapies to be developed for this disease, but also because it will help us gain a better understanding of PD, particularly when associating dementia, and AD.

We have recently performed a large-scale genetic analysis in DLB that showed similarities in common genetic risk between this disease, PD, and AD (Bras et al., 2014) using NeuroX, a genome-wide genotyping array (Nalls et al., 2015). To better understand and quantify these similarities we have now estimated the proportion of variance explained by all single nucleotide polymorphisms of the DLB cohort, and of independent AD and PD cohorts of similar size. We then performed a bivariate restricted maximum likelihood analysis of the genetic relationship matrix, to quantify the genetic covariance between pairs of diseases.

## 2 Methods

Details of the DLB cohort have been published previously (Bras et al., 2014). We used a cohort of 804 European PD cases and a cohort of 959 clinically diagnosed European AD cases, as well as 2806 European and North-American controls, genotyped on Illumina's NeuroX. The PD samples are a UK-only subset of the previously published PD and control dataset (Nalls et al., 2014). The AD cases were diagnosed as either definite or probable AD according to National Institute of Neurological and Communicative Disorders and Stroke and the Alzheimer's disease and Related Disorders Association (McKhann et al., 1984), and the Consortium to Establish a Registry for Alzheimer's disease guidelines (Mirra et al., 1991). All samples used in this study were received with informed consents approved by the local Ethics Committees (Table 1).

Following standard raw data quality control procedures, which included removing variants with GenTrain scores (a metric to assess genotyping quality) lower than 0.9 and samples with call rate lower than 90% (meaning that samples that had less than 90% of the markers genotyped were excluded), we removed markers that had a genotyping rate of >10% and a minor allele frequency of <3%. To generate covariates for the analysis, multidimensional scaling was used to quantify genetic distances between members of the entire cohort.

After estimating the genetic relationship matrix between pairs of individuals, we performed a bivariate restricted maximum likelihood analysis on that matrix, as implemented in the software genome-wide complex trait analysis (Lee et al., 2012, Yang et al., 2010) using the first 2 principal components from multidimensional scaling.

For each comparison the control population was randomized and 1403 controls were assigned to each disease. The analysis between DLB and AD was then repeated excluding markers in the *APOE* region.

## 3 Results

When using the entire array content, after quality control procedures, the estimates for the proportion of variance explained by all single nucleotide polymorphisms for DLB was 0.31 (SE ± 0.03), for AD was 0.6 (SE ± 0.05), and for PD was 0.28 (SE ± 0.05). When excluding the *APOE* region, the estimates were 0.22 (SE ± 0.03), 0.42 (SE ± 0.05), and 0.28 (SE ± 0.05), for DLB, AD, and PD. The decrease seen in DLB and AD reflect the strong and robust association of the *APOE* locus in these diseases.

When comparing pairs of diseases for genetic correlation (i.e., estimating the additive genetic effect i.e., shared between pairs of traits), the highest score was obtained for the AD/DLB pair (0.578, SE ± 0.075). The comparison between PD or DLB yielded a correlation score of 0.362 (SE ± 0.107). Both scores were highly significant with *p*-values of 1.1 × 10^−12^ and 7.1 × 10^−4^, respectively. As a control experiment, we compared AD/PD and obtained a significantly lower score 0.08 (SE ± 0.101) (*p*-value = 0.006, with the most conservative estimate provided by the cocor.dep.groups.overlap function from the cocor package in R, a test of significance for the difference between 2 correlations based on dependent groups with 1 variable in common), that does not deviate from the null hypothesis of no correlation (*p*-value = 0.39).

Given the strong effect from *APOE* in AD and DLB, we have performed the same analysis excluding this locus in these 2 cohorts and obtained a correlation score for AD/DLB_NO_APOE (0.332 ± 0.106) that is not statistically different from the PD/DLB correlation (0.362 ± 0.107) (*p*-value = 0.761, using the same test as mentioned previously). The AD/DLB_NO_APOE correlation is still highly significant 1.8 × 10^−3^ (Table 2).

## 4 Discussion

We have previously described that DLB shares genetic risk determinants with both PD and AD. Here we quantify that overlap by showing that these diseases are, in fact, correlated from a purely genetic perspective.

The DLB cohort is the largest reported so far and a majority of these cases are neuropathologically confirmed (85%), which greatly increases the diagnostic accuracy (Bras et al., 2014). The numbers of PD and AD cases in this study are small, particularly when in comparison with other published datasets. We should note, however, that the fact that we fully replicate the phenotypic variance associated with all types of PD from large meta-analysis studies (Keller et al., 2012), suggests these cohorts are representative, and not substantially underpowered for this type of analysis. In addition, our data shows no genetic correlation between PD and AD (correlation = 0.08, SE ± 0.101, *p*-value = 0.39 when correlation is fixed at 0), a result that replicates previous independent findings (Moskvina et al., 2013).

It should be noted that although being a genome-wide array, NeuroX is not a completely unbiased genotyping platform. A proportion of the variants assayed in this array were included because they were known to be involved in these diseases. Because of this, some of these values may be inflated, however, for the purposes of determining genetic correlation, and comparing between pairs of diseases, this should have no discernible effect.

That DLB seems to share approximately the same amount of genetic risk determinants with PD and AD fits with our understanding of this disease, given the clinical and neuropathological overlap. Although not assessed in this work, it would be interesting to test if these correlations reflect quantitative pathology (e.g., would excluding DLB cases with prominent AD-related pathology reduce the correlation score between DLB and AD).

## 5 Conclusions

This is the first study to look at genetic correlation between DLB, PD, and AD. Despite using small cohorts, we show that these data replicate previously published results. We also show that DLB shares approximately the same amount of genetic determinants with PD as it does with AD, when the *APOE* locus is excluded. These results show us that, from a mechanistic standpoint, DLB is a different, but highly related disease to both AD and PD. They further emphasize the need for more studies in DLB—this is a greatly underappreciated disease and these data strongly support this fact. Fully dissecting the genetic architecture of DLB will allow us to gain a better understanding of not just one but all 3 diseases. In addition, these data also show that we should gradually move from the current model of binary diagnosis to a more quantitative one.

## Disclosure statement

The authors have no conflicts of interest to disclose.

## Figures and Tables

**Table 1:** Samples included in the study

Trait	Total cases	Pathologically confirmed cases
DLB	788	667
AD	959	113
PD	804	0

Key: AD, Alzheimer's disease; DLB, dementia with Lewy bodies; PD, Parkinson's disease.

**Table 2:** Genetic correlation scores between pairs of diseases

Trait1	Trait2	Genetic correlation	SE	*p*-value
AD	DLB	0.578	0.075	1.1 × 10^−12^
PD	DLB	0.362	0.107	7.1 × 10^−4^
AD	PD	0.08	0.101	0.39
AD	DLB_NO_APOE	0.332	0.106	1.8 × 10^−3^

Key: AD, Alzheimer's disease; DLB, dementia with Lewy bodies; PD, Parkinson's disease; SE, standard error.
